# A Comprehensive Analysis of the Glutathione Peroxidase 8 (GPX8) in Human Cancer

**DOI:** 10.3389/fonc.2022.812811

**Published:** 2022-03-25

**Authors:** Zhijing Ren, Yu He, Qinqin Yang, Jiajia Guo, Haifeng Huang, Bo Li, Dong Wang, Zhen Yang, Xiaobin Tian

**Affiliations:** ^1^ Department of Clinical Laboratory, Guizhou Provincial People’s Hospital, Guiyang, China; ^2^ Medical College, Guizhou University, Guiyang, China; ^3^ Department of Orthopedics, Guizhou Provincial People’s Hospital, Guiyang, China; ^4^ Department of Orthopedics, Affiliated Cancer Hospital of Guizhou Medical University, Guiyang, China; ^5^ Department of Orthopedics, Affiliated Hospital of Guizhou Medical University, Guiyang, China

**Keywords:** GPX8, pan-cancer, prognosis, immune infiltration, GBM

## Abstract

**Objective:**

Nowadays, cancer is still a leading public health problem all over the world. Several studies have reported the GPX8 could be correlated with the poor prognostic of Gastric Cancer and Breast Cancer. However, the prognostic potential of GPX8 in pan-cancer remains unclear. In this work, we aimed to explore the prognostic and immunological role of GPX8 in human cancer and confirm the oncogenic value in GBM.

**Methods:**

The data of TCGA, CPTAC and GEO databases were adopted for the survival analysis. Based on the RNAseq and Methylation450 data of TCGA, the R language and package “ggplot2” were used to analyze the DNA methylation at the region of the promoter of GPX8 in tumors. The genetic alteration of GPX8 from TCGA cancers was investigated in cBioPortal. The R package “GSVA” and “ssGSEA” were employed to evaluate the correlation of GPX8 expression with the immune infiltration. The KEGG website was used for pathway analysis. The STRING website and GEPIA were performed to predict GPX8-binding proteins. The R package “ggplot2” and “clusterprofile” were used to analyze and visualize the GO and KEGG analysis. A normal human astrocyte cell line and three GBM cell lines were cultured under suitable conditions. The shRNA was transferred to cells by Lipofectamine 3000. The qRT-PCR and WB were adopted to detect the expression of GPX8. The wound-healing assay and transwell assay were taken to analyze the invasive and metastatic abilities. The tumor tissues and paracancerous ones were collected from patients with GBM. WB assay was employed to analyze the expression of GPX8 protein.

**Results:**

GPX8 was a valuable diagnostic biomarker in multiple cancers, including GBM/LGG (glioblastoma multiforme/Brain lower grade glioma), KIRC (kidney renal clear cell carcinoma), KIRP (kidney renal papillary cell carcinoma) and STAD (stomach adenocarcinoma). Moreover, we observed a correlation between the expression of GPX8 and the reduced DNA methylation at the promoter region in several tumors, such as GBM/LGG. Our results indicated a positive correlation between the GPX8 expression and immune infiltration. In addition, the enrichment analysis demonstrated that antioxidant activity was mainly involved in the functional mechanism of GPX8. In particular, we first confirmed the up-regulated of GPX8 in GBM cells and observed the suppression of migrative and invasive phenotypes by knockdown of GPX8. Furthermore, we confirmed the expression of GPX8 was higher in GBM tumor tissues than paracancerous ones.

**Conclusion:**

Our study showed a correlation of GPX8 expression with clinical prognosis, DNA methylation and immune infiltrates. Furthermore, we first confirmed GPX8 was highly expressed in GBM cells and contributed to migration and invasion. These results provided a predictive biomarker and an inclusive understanding of the GPX8 expression in multiple tumors types, especially in GBM.

## Introduction

Nowadays, cancer is a main public health problem all over the world. Partly because of the benefit of significant development in the recognition, diagnosis, and treatment, the mortality ratio has improved in many common cancer types (1). However, compared to the UK and USA, China has a larger scale of cancer types with a poorer 5-year overall survival rate. The mortality and incidence ratio in patients with cancer is still extremely high (2, 3). Therefore, it is necessary to search for new biomarkers and treatment targets for cancers.

Since tumorigenesis is a complex and variable process, multiple genetic changes could impact the malignant transformation and clinical outcomes in cancer patients (4, 5). The pan-cancer expression analysis, which contains massive data from publicly funded databases, has been deemed an essential and effective means for researchers to analyze the correlation between gene expression and clinical prognosis. For example, previous studies have demonstrated that the SND1, HER2 and LAYN could act as predictive biomarkers for cancer prognosis (6–8).

It is generally accepted that the immune system activated by tumor cells could cause immune surveillance and defense to inhibit tumor progression (9). Nevertheless, as a double-edged sword, more and more evidence indicates that the multiple immune cells recruited by progressed tumors could stimulate an inflammatory environment and promote the secretion of cytokines and chemokines, which contribute to tumor growth, metastasis, invasion and pathological angiogenesis (10–12). Research of immune-related mechanisms is a promising direction and could prompt the development of cancer treatment (2). For instance, the programmed death-1 (PD-1), an immune checkpoint molecule, was been recognized as a target for cancer treatment (13). Multiple medicines targeted PD-1 have been approved by the FDA for the usage in malignant melanoma (13–15). Moreover, the tumor-infiltrating Immune cells have been verified to affect the prognosis (16).

Glutathione peroxidases (GPxs) (EC 1.11.1.9) are a kind of enzyme family that could protect organisms from oxidative damage by the reactive oxygen species (ROS) and has an important influence on substance metabolism (17–20). As a member of the GPX family, GPX8 is a transmembrane protein localized in the ER (21). It has been shown GPX8 can reduce oxidized PDI and prevent Ero1α-derived H2O2 leaking by interacting with Ero1α, a member of the sulfhydryl oxidase Ero1 family (21, 22). Increasing evidence has verified that GPX8 could be correlated with the poor prognostic of cancers and involved in diverse physiological processes and tumorigenesis, including gastric cancer, lung cancer and others (23–25). Furthermore, reports have indicated that ROS could modulate the function of immune cells (26–28). However, although the emerging genes are related to the prognosis, we have never retrieved any report with a pan-cancer analysis of GPX8. Thus, in this work, we aimed to explore the prognostic and immunological role of GPX8 in human cancer and confirm the oncogenic value in GBM.

## Materials and Methods

### GPX8 Expression in Human Cancers

The expression of GPX8 gene in different types of cancers was explored in the TIMER database (http://timer.cistrome.org/) and ONCOMINE database (www.oncomine.org) with the conditions: fold change > 2 and P < 0.001. The R language (version 3.6.3) and R package “ggplot2” were performed to analyze and visualize the RNAseq data of TCGA processed uniformly by the toiling process (29). The CPTAC analysis of the UALCAN portal (http://ualcan.path.uab.edu/analysis-prot.html) was used to identify the protein expression level of GPX8 in six cancers, including breast cancer, colon cancer, clear cell RCC (Renal cell carcinoma), LUAD (Lung adenocarcinoma), UCEC (Uterine corpus endometrial carcinoma) and ovarian cancer. The Human Protein Atlas (HPA) (http://www.proteinatlas.org/) were employed to confirm the expression of GPX8 in normal or tumor tissue of colon, kidney, lung, endometrium.

### Survival and ROC Curve Analysis

The TCGA datasets were employed to investigate the correlation of GPX8 expression with the prognosis of different cancers. The Kaplan-Meier Plotter (https://kmplot.com/analysis/) was performed to analyze the correlation between GPX8 expression and the Overall Survival and Disease Specific Survival. The PrognoScan (http://dna00.bio.kyutech.ac.jp/PrognoScan/index.html) was adopted to analyze the correlation between GPX8 expression and survival in GSE4412-GPL97, GSE4271-GPL97, GSE17536 and GSE31210. The R package “pROC” package and “ggplot2” were used to analyze and visualize the RNAseq data of TCGA and GTEx. The area under the ROC curves (AUC) was calculated for the determination of diagnosis and prognosis.

### DNA Methylation and Genetic Alteration Analysis

To analyze and visualize the RNAseq and Methylation450 data of TCGA, the R language and package “ggplot2” were used. Moreover, the genetic alteration of GPX8 from TCGA cancers was explored in cBioPortal (https://www.cbioportal.org/) and displayed as alteration frequency.

### Immune Infiltration Analysis

The “Gene_Corr” module of TIMER2 was used to explore the correlation between GPX8 and cancer-associated fibroblasts. The single sample gene set enrichment analysis (ssGSEA) of R package “GSVA” (30) was used to analyze the correlation between GPX8 and Immune Cells, including aDC [activated dendritic cells], B cells, CD8 T cells, Cytotoxic cells, DC [dendritic cells]; Eosinophils, iDC [immature dendritic cells], Macrophages, Mast cells, Neutrophils, NK CD56bright cells, NK CD56dim cells, NK cells, pDC [Plasmacytoid dendritic cells], T cells, T helper cells, Tcm [T central memory], Tem [T effector memory], Tfh [T follicular helper], Tgd [T gamma delta], Th1 cells, Th17 cells, Th2 cells and Treg.

### GPX8-Related Gene Enrichment Analysis

The KEGG website (https://www.kegg.jp/) was used for pathway analysis. The STRING website (https://string-db.org/) and GEPIA (http://gepia.cancer-pku.cn/index.html) were performed to predict GPX8-binding proteins. The R package “ggplot2” and “clusterProfiler” (31) were used to analyze and visualize the GO and KEGG analysis. The GBM/LGG, KIRC, KIRC and STAD project RNAseq data from TCGA (https://portal.gdc.cancer.gov/) were applied to analyze the co-expression among GPX8, IKBIP, SERPINH1, PPIC, OSTC, TNFAIP8 and CRTAP.

### Patients and Tissue Samples

GBM tissues and paracancerous ones were surgically resected from 3 patients undergoing surgery or biopsy, which were pathologically confirmed and stored at −80°C. This study got approval from the Ethics Committee of Guizhou Provincial People’s Hospital and was conducted after obtaining written informed consent of each subject.

### Cell Culture and Transfection

A normal human astrocyte cell line (HA) was received from ScienCell (Carlsbad, USA) and maintained in Astrocyte Medium. A GBM cell line LN-299 was received from ATCC (Rockville, USA). The other two GBM cell lines A172 and U251 were received from the cell bank of the Chinese Academy of Science (Shanghai, China). These cells were maintained in DMEM medium with 10% FBS and cultured in suitable conditions (37°C; 5% CO2). All shRNAs (sh-NC,sh-GPX8-1 and sh-GPX8-2) were purchased from General Biosystems (Anhui, China) and transfected to cells by Lipofectamine 3000 (Invitrogen, USA). The Sequences of shRNAs were displayed in **Table S1**.

### Quantitative Reverse Transcription-PCR (qRT-PCR)

Cells were treated with Trizol (Takara, Japan). Total RNA was reverse transcribed by the RT kit (Takara, Japan). SYBR Green PCR kit (Takara, Japan) was performed for qRT-PCR. The relative expression of GPX8 was analyzed by the 2^-△△Ct^ method and normalized with GAPDH. The primer sequences were exhibited in **Table S2**.

### Western Blot Assay (WB)

Cells and tissue samples were collected for protein extraction. The BCA Protein assay kit (Beyotime, China) was used for protein concentration evaluation. Proteins were separated with a sodium dodecyl sulfate–polyacrylamide gel electrophoresis gel. The polyvinylidene fluoride membrane containing proteins was blocked with 5% milk. Then, specific primary antibodies (GPX8, Abcam; GAPDH, Abcam) were applied to the membrane at 4°C overnight. After incubating with secondary antibodies, an ECL Western Blotting Substrate (Solarbio, China) was used to detect the protein blots.

### Wound-Healing Assay

LN-299 and U251 cells were cultured in plates for 24h. The pipette tip was employed to draw the surface of the cell layer. The microscope was used to capture the images at 0 and 48h after injury. The distance of the injury area at 48h was measured. A relative migration rate was calculated by normalizing the distance of the injury area at 0h.

### Transwell Assay

Transwell chamber with matrigel was performed to transwell assay. Cells were mixed with serum-free media and injected into the upper layer. Outside of the transwell chamber was full with complete medium. After cells were cultured in suitable conditions for 48h, the transwell chambers were taken for fixing and staining by 4% paraformaldehyde and 0.1% crystal violet (Solarbio, China). The cells on the bottom of the chambers were counted.

### Statistical Analysis

SPSS 22.0 (IBM, USA) was used for statistical analyses. The data were analyzed with the Student’s t-test or one-factor analysis. All results were displayed as mean ± SD. *P <*0.05 was regarded as statistically significant.

## Results

### GPX8 Expression in Pan-Cancer

**Figure 1:**
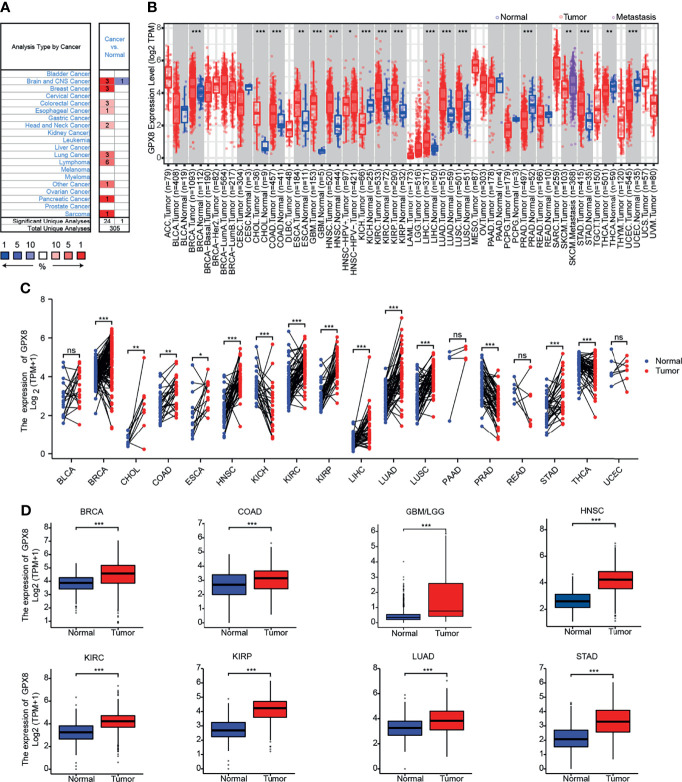
The expression of GPX8 gene in different cancers. **(A)** The expression of GPX8 in pan-cancer was explored in the Oncomine database. (www.oncomine.org). **(B)** The expression of GPX8 in pan-cancer was explored in TCGA through TIMER. (http://timer.cistrome.org/). **(C)** The expression of GPX8 in pan-cancer was analyzed using paired tumor/normal samples from TCGA and GTEx databases. **(D)** The expression of the GPX8 was analyzed in BRCA, COAD, GBM/LGG, HNSC, KIRC, KIRP, LUAD and STAD. *P < 0.05, **P < 0.005, ***P < 0.001. “ns” was regarded as no statistically significant.

**Figure 2:**
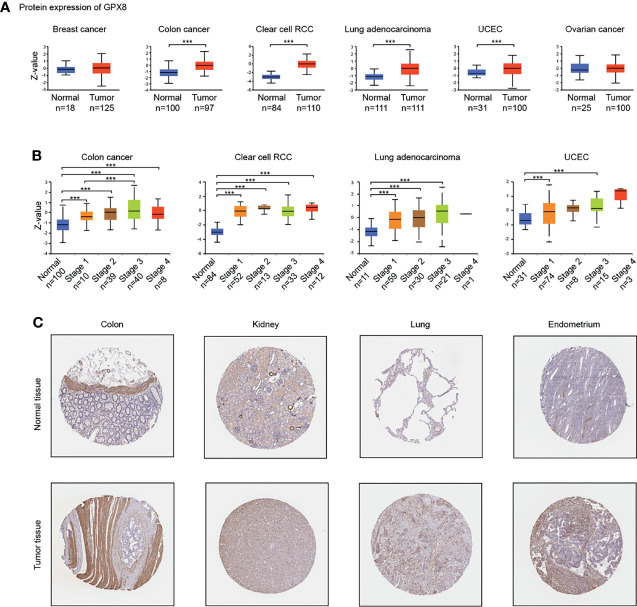
The expression of GPX8 protein in different cancers. **(A)** Based on the CPTAC analysis (http://ualcan.path.uab.edu/analysis-prot.html), the expression of GPX8 protein was analyzed in Breast cancer, colon cancer, clear cell RCC, Lung adenocarcinoma, UCEC, Ovarian cancer. **(B)** The expression of GPX8 protein was analyzed by the main pathological stages of colon cancer, clear cell RCC, Lung adenocarcinoma, UCEC. **(C)** Based on the HPA database (http://www.proteinatlas.org/), the expression of GPX8 protein in normal or tumor tissue of colon, kidney, lung, the endometrium was displayed. ***P < 0.001.

To ascertain differences in GPX8 expression between the tumor and normal tissues, several databases were used, including Oncomine, TCGA, GEO and others. By searching in the Oncomine, the expression level of the GPX8 gene was found significantly up-regulated in many types of cancers, including BRCA (Breast invasive carcinoma), CHOL(Cholangiocarcinoma), COAD (Colon adenocarcinoma), GBM(Glioblastoma multiforme), HNSC(Head and Neck squamous cell carcinoma), KIRC(Kidney renal clear cell carcinoma), KIRP(Kidney renal papillary cell carcinoma), LIHC(Liver hepatocellular carcinoma), LUAD(Lung adenocarcinoma), LUSC(Lung squamous cell carcinoma), STAD(Stomach adenocarcinoma) **(** **Figures 1A, B** **)**. The tumor and adjacent normal tissues for GPX8 across all TCGA tumors were shown in **Figure 1C**. After including the normal tissue of the GTEx dataset as controls, we further confirmed the GPX8 expression was significantly higher in BRCA, COAD, GBM/LGG, HNSC, KIRC, KIRP, LUAD and STAD compared with normal tissues **(** **Figure 1D** **)**. Moreover, the result of CPTAC analysis demonstrated the protein expression of GPX8 was up-regulated and correlated with the pathological stages of colon cancer, clear cell RCC, LUAD and UCEC **(** **Figures 2A, B** **)**. The data of the HPA database showed the different expression levels of GPX8 in normal or tumor tissue of the colon, kidney, lung, endometrium **(** **Figure 2C** **)**. In a word, GPX8 was highly expressed in most cancers.

### Prognostic Potential of GPX8 in Cancers

**Figure 3:**
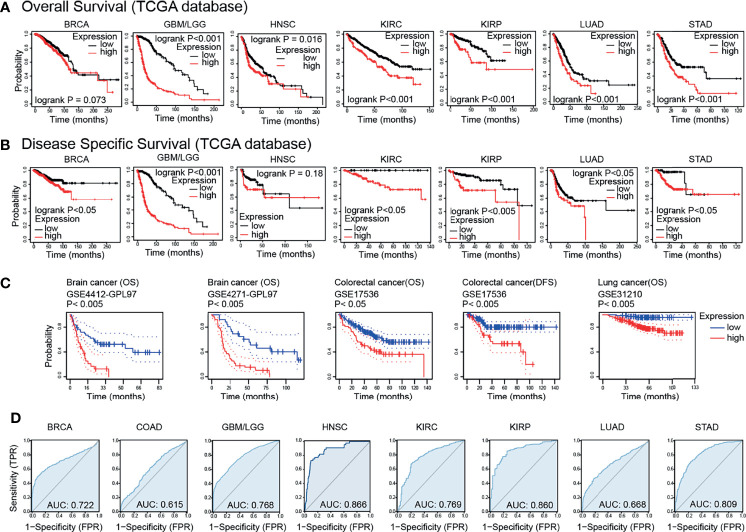
Survival prognosis analysis of cancers. **(A)** Overall Survival analysis of GPX8 genes in the TCGA dataset. (https://kmplot.com/analysis/). **(B)** Disease Specific Survival analysis of GPX8 genes in the TCGA dataset. (https://kmplot.com/analysis/). **(C)** Survival analysis of GPX8 genes in GSE4412-GPL97, GSE4271-GPL97, GSE17536 and GSE31210 (http://dna00.bio.kyutech.ac.jp/PrognoScan/index.html). **(D)** ROC analysis of GPX8 genes in TCGA database.

Based on the TCGA databases, the results of survival analysis demonstrated that the GPX8 expression significantly affects prognosis in GBM/LGG, KIRC, KIRP, LUAD and STAD (**Figures 3A, B**). Moreover, the GEO datasets, including GSE4412-GPL97, GSE4271-GPL97, GSE17536 and GSE31210, showed that high GPX8 expression was correlated with poorer prognosis of patients with Brain cancer, Colorectal cancer and Lung cancer (**Figure 3C**). Furthermore, the receiver operating characteristic (ROC) curve was emplored to verify the diagnostic value of GPX8 in different cancers. As shown in **Figure 3D**, the GPX8 had a moderate diagnostic accuracy of BRCA, GBM/LGG, HNSC, KIRC, KIRP and STAD (AUCs were above 0.7 and even 0.8). To summarize, the Survival and ROC curve analyses indicated that GPX8 was a valuable diagnostic biomarker in multiple types of cancers, including GBM/LGG, KIRC, KIRP and STAD.

### Correlation Between GPX8 Expression and Critical Clinical/Pathological Features

**Table 1** provided the relations between clinical data and GPX8 expression in GBM/LGG, KIRC, KIRP and STAD. High GPX8 expression was correlated with the WHO grade and age of GBM/LGG, and the T stage and Pathologic stage of KIRC and STAD. However, the high expression of GPX8 was not significantly correlated with other clinical features of KIRP. Univariate analysis demonstrated that the OS was correlated with the WHO grade and T stage of GBM/LGG, the N stage, M stage and the Pathologic T stage of KIRC and KIRP and the N stage and M stage of STAD. In multivariate analysis, the OS was associated with the WHO grade of GBM/LGG, the M stage of KIRC, KIRP and STAD and the N stage of STAD (**Table 2**).

**Table 1:** Correlation of the GPX8 expression and the clinical characteristic of patients with cancers.

Characteristic	GBM/LGG			KIRC			KIRP			STAD		
	Low	High	*p*	Low	High	*p*	Low	High	*p*	Low	High	*P*
n	348	348		269	270		144	145		187	188	
WHO grade, n (%)			**<0.001**			/						/
G2	183 (28.8%)	41 (6.5%)		/	/		/	/		/	/	
G3	118 (18.6%)	125 (19.7%)		/	/		/	/		/	/	
G4	1 (0.2%)	167 (26.3%)		/	/		/	/		/	/	
T stage, n (%)						**0.026**			0.061			**0.002**
T1	/	/		150 (27.8%)	128 (23.7%)		104 (36.2%)	89 (31%)		17 (4.6%)	2 (0.5%)	
T2	/	/		40 (7.4%)	31 (5.8%)		16 (5.6%)	17 (5.9%)		44 (12%)	36 (9.8%)	
T3	/	/		76 (14.1%)	103 (19.1%)		22 (7.7%)	37 (12.9%)		83 (22.6%)	85 (23.2%)	
T4	/	/		3 (0.6%)	8 (1.5%)		0 (0%)	2 (0.7%)		43 (11.7%)	57 (15.5%)	
N stage, n (%)			/			0.050			0.073			0.191
N0	/	/		114 (44.4%)	127 (49.4%)		24 (31.2%)	25 (32.5%)		66 (18.5%)	45 (12.6%)	
N1	/	/		3 (1.2%)	13 (5.1%)		7 (9.1%)	17 (22.1%)		47 (13.2%)	50 (14%)	
N2	/	/		/	/		0 (0%)	4 (5.2%)		35 (9.8%)	40 (11.2%)	
N3	/	/		/	/		/	/		34 (9.5%)	40 (11.2%)	
M stage, n (%)			/			0.603			1.000			0.230
M0	/	/		209 (41.3%)	219 (43.3%)		45 (43.3%)	50 (48.1%)		167 (47%)	163 (45.9%)	
M1	/	/		35 (6.9%)	43 (8.5%)		4 (3.8%)	5 (4.8%)		9 (2.5%)	16 (4.5%)	
Pathologic stage, n (%)			/			**0.039**			0.075			**0.003**
Stage I	/	/		149 (27.8%)	123 (22.9%)		92 (35.4%)	80 (30.8%)		39 (11.1%)	14 (4%)	
Stage II	/	/		33 (6.2%)	26 (4.9%)		10 (3.8%)	12 (4.6%)		54 (15.3%)	57 (16.2%)	
Stage III	/	/		50 (9.3%)	73 (13.6%)		17 (6.5%)	34 (13.1%)		72 (20.5%)	78 (22.2%)	
Stage IV	/	/		37 (6.9%)	45 (8.4%)		6 (2.3%)	9 (3.5%)		15 (4.3%)	23 (6.5%)	
Age, mean ± SD	38 (31, 48)	55 (42.75, 63)	**<0.001**	60.81 ± 11.93	60.45 ± 12.27	0.731	62.83 ± 11.46	60.31 ± 12.23	0.073	65.91 ± 10.95	65.75 ± 10.37	0.888

The meaning of the bold values was regarded as statistically significant.

**Table 2:** Univariate and multivariate analyses of overall survival.

Cancer type	Characteristics	Total (N)	Univariate analysis		Multivariate analysis	
			Hazard ratio (95% CI)	*P* value	Hazard ratio (95% CI)	*P* value
GBM/LGG	WHO grade (G3&G4 *vs.* G2)	634	5.642 (3.926-8.109)	**<0.001**	2.411 (1.556-3.735)	**<0.001**
KIRC	T stage (T2&T3&T4 *vs.* T1)	539	2.917 (2.095-4.061)	**<0.001**	1.340 (0.811-2.212)	0.253
	N stage (N1 *vs.* N0)	257	3.453 (1.832-6.508)	**<0.001**	1.788 (0.927-3.450)	0.083
	M stage (M1 *vs.* M0)	506	4.389 (3.212-5.999)	**<0.001**	3.403 (2.108-5.496)	**<0.001**
KIRP	Pathologic T stage (T2&T3&T4 *vs.* T1)	286	5.074 (2.637-9.765)	**<0.001**	0.828 (0.165-4.161)	0.819
	Pathologic N stage (N1&N2 *vs.* N0)	77	5.003 (2.062-12.140)	**<0.001**	1.819 (0.396-8.350)	0.442
	Pathologic M stage (M1 *vs.* M0)	104	114.966 (22.481-587.925)	**<0.001**	51.689 (4.901-545.151)	**0.001**
STAD	T stage (T3&T4 *vs.* T1&T2)	362	1.719 (1.131-2.612)	**0.011**	1.450 (0.919-2.290)	0.111
	N stage (N1&N2&N3 *vs.* N0)	352	1.925 (1.264-2.931)	**0.002**	1.661 (1.058-2.607)	**0.028**
	M stage (M1 *vs.* M0)	352	2.254 (1.295-3.924)	**0.004**	1.866 (1.057-3.293)	**0.031**

The meaning of the bold values was regarded as statistically significant.

### The Expression of GPX8 Was Correlated to DNA Methylation and Genetic Alteration

**Figure 4:**
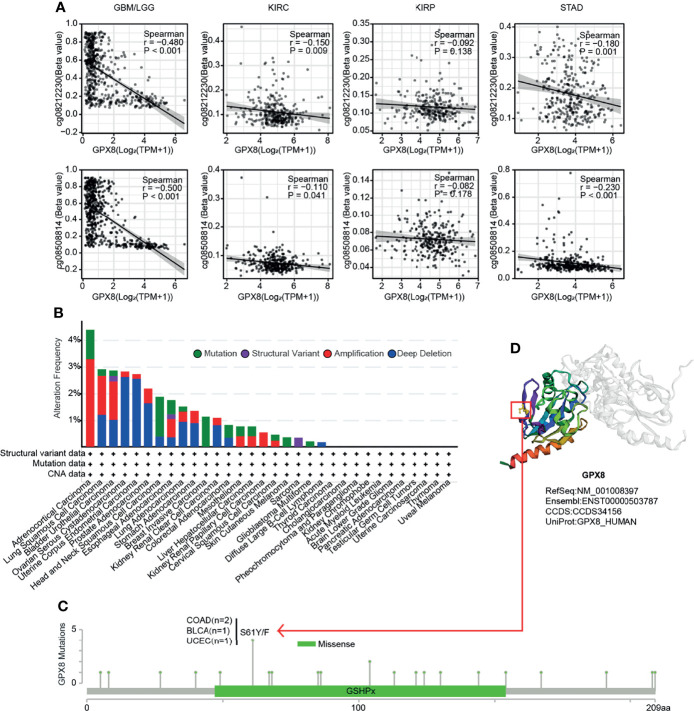
DNA methylation analysis and Mutation feature of GPX8 in cancers. **(A)** DNA methylation of GPX8 in BM/LGG, KIRC, KIRP and STAD. **(B)** The alteration frequency and mutation type of GPX8. (https://www.cbioportal.org/). **(C)** The alteration frequency with mutation type and mutation site are displayed. (https://www.cbioportal.org/). **(D)** The mutation site with the highest alteration frequency (S61Y/F) in the 3D structure of GPX8. (https://www.cbioportal.org/).

Through exploring in the RNAseq and Methylation450 data of TCGA, a negative correlation between GPX8 expression and GPX8 DNA methylation of the promoter region was observed in GBM/LGG, KIRC and STAD (**Figure 4A**). However, the different cancer types showed a different correlation coefficient. For instance, the correlation coefficient of two probes (cg08212230 and cg08508814) were -0.480 and -0.5 in GBM/LGG and were 0.15 and 0.11 in KIRC. Furthermore, a genetic alteration analysis showed the alteration frequency of GPX8 was >4%, and the primary type was amplification (**Figure 4B**). Moreover, based on the cbioportal database, the missense mutation of GPX8 has been found as the main type of genetic alteration, and S61Y/F alteration was the important site, which was detected in 2 cases of COAD, 1 case of BLCA and 1 case of UCEC **(** **Figure 4C** **)**. The S61Y/F site in the 3D structure of GPX8 protein was shown in **Figure 4D**.

### Immune Infiltration Analysis

**Figure 5:**
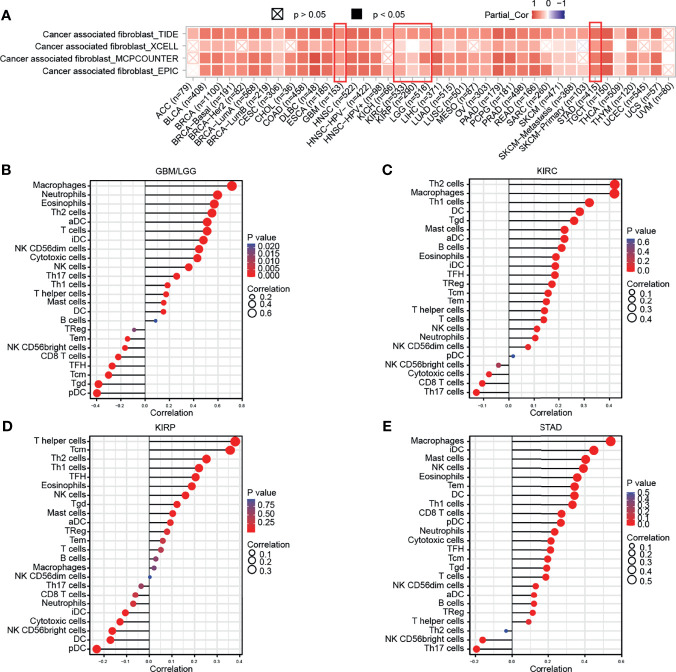
Correlation analysis between GPX8 expression and tumor-infiltrating immune cells. **(A)** Correlation analysis between GPX8 expression and CAFs in pan-cancer (http://timer.comp-genomics.org/). **(B–E)** Correlation analysis between GPX8 expression and immune cells in GBM/LGG, KIRC, KIRP, STAD.

**Figure 6:**
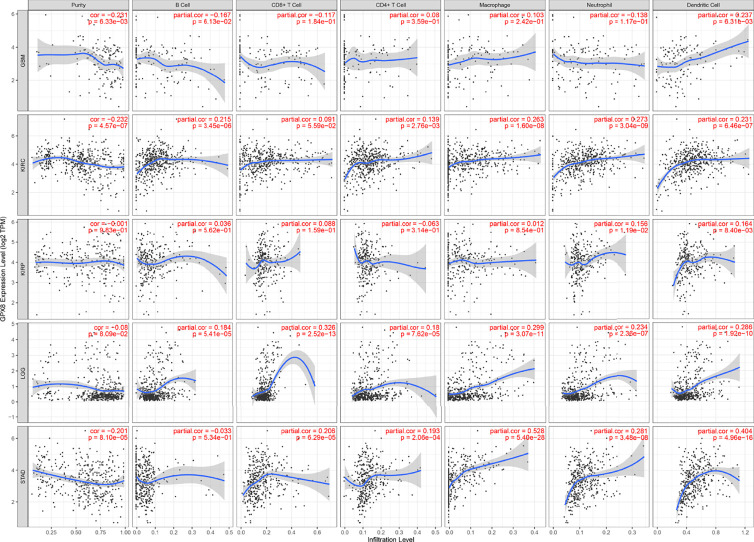
Correlation analysis between GPX8 expression and immune cells in TIMER (http://timer.cistrome.org/).

Subsequently, the results of TIMER, TIDE, XCEL, MCPCOUNTER, EPIC algorithms indicated a positive correlation between the GPX8 expression and cancer-associated fibroblast in many human cancers, including GBM, LGG, KIRC, KIRP and STAD (**Figure 5A**). Moreover, based on the TCGA database, the result of R package “GSVA” was demonstrated the positive correlation of GPX8 and the different immune cells was observed in these cancers (**Figures 5B–E**), and the detail was displayed in **Table 3**. Furthermore, the scatterplot data from the TIMER database indicated that the GPX8 expression level was correlated with immune cells (**Figure 6**). To sum up, the expression of GPX8 was related to Immune infiltration.

**Table 3:** Correlation analysis between GPX8 and immune cells.

Cells	GBM/LGG		KIRC		KIRP		STAD	
	Cor	*P*	Cor	*P*	Cor	*P*	Cor	*P*
aDC	0.511	**<0.001**	0.222	**<0.001**	0.093	0.114	0.119	**0.021**
B cells	0.086	**0.023**	0.211	**<0.001**	0.029	0.625	0.119	**0.021**
CD8 T cells	-0.221	**<0.001**	-0.105	**0.014**	-0.061	0.301	0.272	**<0.001**
Cytotoxic cells	0.430	**<0.001**	-0.079	0.068	-0.129	**0.028**	0.215	**<0.001**
DC	0.151	**<0.001**	0.282	**<0.001**	-0.172	**0.003**	0.343	**<0.001**
Eosinophils	0.570	**<0.001**	0.188	**<0.001**	0.188	**0.001**	0.358	**<0.001**
iDC	0.480	**<0.001**	0.186	**<0.001**	-0.106	0.072	0.448	**<0.001**
Macrophages	0.715	**<0.001**	0.419	**<0.001**	0.021	0.720	0.540	**<0.001**
Mast cells	0.153	**<0.001**	0.222	**<0.001**	0.104	0.078	0.404	**<0.001**
Neutrophils	0.597	**<0.001**	0.104	**0.016**	-0.071	0.229	0.235	**<0.001**
NK CD56bright cells	-0.166	**<0.001**	-0.041	0.341	-0.163	**0.005**	-0.161	**0.002**
NK CD56dim cells	0.445	**<0.001**	0.076	0.079	0.003	0.962	0.128	**0.013**
NK cells	0.359	**<0.001**	0.111	**0.010**	0.160	**0.006**	0.391	**<0.001**
pDC	-0.397	**<0.001**	0.017	0.698	-0.232	**<0.001**	0.269	**<0.001**
T cells	0.511	**<0.001**	0.140	**0.001**	0.051	0.385	0.187	**<0.001**
T helper cells	0.173	**<0.001**	0.143	**<0.001**	0.380	**<0.001**	0.090	0.082
Tcm	-0.301	**<0.001**	0.158	**<0.001**	0.358	**<0.001**	0.197	**<0.001**
Tem	-0.145	**<0.001**	0.151	**<0.001**	0.060	0.311	0.344	**<0.001**
TFH	-0.272	**<0.001**	0.184	**<0.001**	0.205	**<0.001**	0.212	**<0.001**
Tgd	-0.385	**<0.001**	0.260	**<0.001**	0.123	**0.037**	0.192	**<0.001**
Th1 cells	0.184	**<0.001**	0.321	**<0.001**	0.220	**<0.001**	0.332	**<0.001**
Th17 cells	0.260	**<0.001**	-0.129	**0.003**	-0.036	0.543	-0.194	**<0.001**
Th2 cells	0.551	**<0.001**	0.420	**<0.001**	0.253	**<0.001**	-0.033	0.523
TReg	-0.091	**0.016**	0.173	**<0.001**	0.079	0.182	0.112	**0.030**

The meaning of the bold values was regarded as statistically significant.

### Enrichment Analysis of GPX8-Related Partners

**Figure 7:**
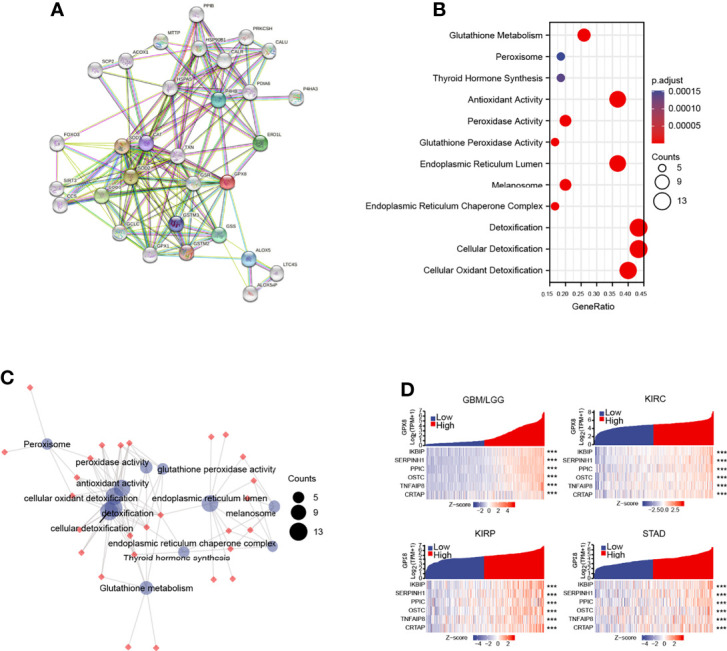
The analysis of GPX8 and GPX8-related partners. **(A)** The GPX8-binding PPI analysis of GPX8. (https://string-db.org/, http://gepia.cancer-pku.cn/index.html). **(B)** The GO and KEGG analysis of GPX8 and GPX8-related partners. (https://www.kegg.jp/). **(C)** The relationship of pathways. **(D)** The co-expression analysis of GPX8 and GPX8-related genes in GBM/LGG, KIRC, KIRP and STAD. (https://portal.gdc.cancer.gov/). ***P < 0.001.

The GPX8-binding PPI networks were created by the STRING online database (**Figure 7A**). The GO and KEGG analysis demonstrated that GPX8 and GPX8-binding proteins were mainly involved in Glutathione metabolism (**Figure 7B**). The relationship of these pathways was displayed (**Figure 7C**). The GBMLGG, KIRC, KIRC and STAD project RNAseq data from TCGA demonstrated that GPX8 was co-expression among IKBIP, SERPINH1, PPIC, OSTC, TNFAIP8 and CRTAP (**Figure 7D**).

### GPX8 Was Highly Expressed and Influenced the Migration and Invasion of GBM Cells

**Figure 8:**
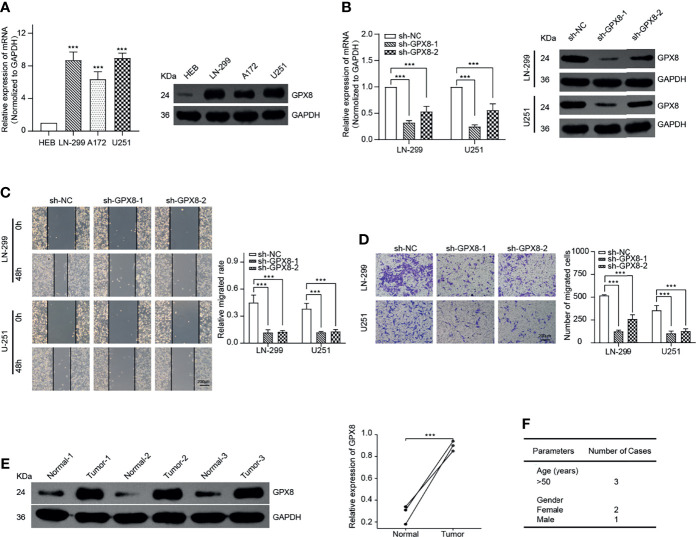
GPX8 is up-regulated in GBM cells and related to migration and invasion *in vitro*. **(A)** qRT-PCR and WB confirmed the expression of GPX8 was higher in GBM cells. **(B)** qRT-PCR and WB verified the efficiency of sh-RNAs. **(C)** Wound-healing assays indicated the knockdown of GPX8 could restrain the migration of GBM cells. **(D)** Transwell assays showed the knockdown of GPX8 could inhibit the invasion of GBM cells. **(E)**. WB confirmed the expression of GPX8 was higher in GBM tumor tissues than paracancerous ones. **(F)** The clinical parameters of the patients. Data were presented as mean ± SD. ***P < 0.001.

To confirm the function of GPX8, we selected GBM as a typical type of human cancer for further investigation. By comparing with the normal human astrocyte cell line, the result of qRT-PCR and WB demonstrates that GPX8 was highly expressed in GBM cells, including LN-299, A172 and U251 (**Figure 8A**). Then, we designed two shRNAs that targeted different sites of GPX8. The result of qRT-PCR and WB indicated both two sh-RNAs have excellent efficiency in knockdown of the GPX8 expression (**Figure 8B**). Subsequently, we observed the knockdown of GPX8 could inhibit the migration and invasion of GBM cells (**Figures 8C, D** **)**. To confirm the expression of GPX8 in primary tumors, we collected the tumor tissues and paracancerous ones from patients with GBM. By comparing with the normal tissue of the same patient, the result of WB implied that GPX8 was highly expressed in GBM tissue **(** **Figure 8E** **)**. The clinical parameters of the patients were displayed in **Figure 8F**.

## Discussion

In this work, we comprehensively investigated the molecular characteristics of the GPX8 gene in 33 different cancers from diverse databases, including TCGA, CPTAC and GEO, and aimed to explore the value of GPX8 in cancer prognosis, progression, and treatment.

Based on our results, compared to normal tissues, the mRNA of GPX8 was highly expressed in multiple types of cancer, especially in BRCA, COAD, GBM/LGG, HNSC, KIRC, KIRP, LUAD and STAD. Furthermore, the UALCAN database data confirmed that the protein expression level of GPX8 in tumor tissues of COAD, KIRC and LUAD were higher than normal, and the higher expression of GPX8 was related to the late clinical stage. Nevertheless, we failed to find significant differences between tumor and normal tissues in Breast cancer. Additionally, although the GPX8 protein was highly expressed in UCEC, the mRNA of GPX8 was lower than normal. As proteins could be modulated at multiple levels, the mRNA/protein expression is not always consistent (32). And the different data sources of mRNA and protein might lead to these differences.

Several studies have reported the GPX8 could be correlated with the poor prognostic of Gastric Cancer and Breast Cancer (24, 33). Herein, based on GEO, TCGA and GTEx data, we conducted a series of survival analyses which found the prognostic value of GPX8 seems to differ for different tumors. For GBM/LGG, KIRC, KIRP and STAD, the GPX8 expression was related to the prognosis of OS and DSS and had good diagnostic accuracy (AUC>0.7). For BRCA and HNSC, there was no significant correlation between GPX8 and OS or DSS. Thus, although the prognostic value of GPX8 in BRCA and HNSC is still debated, the current evidence could suggest the role of GPX8 in the clinical prognosis of GBM/LGG, KIRC, KIRP and STAD.

For cancers, DNA methylation is a crucial mechanism for epigenetic changes. Reports have demonstrated that abnormal DNA methylation has been involved in multiple types of tumorigenesis (34, 35). For example, hypermethylated FBXL7 is often observed in aggressive tumors (36). In our work, evidence suggested that the expression of GPX8 was correlated to the reduced DNA methylation at the promoter region in different types of tumors, especially for patients with GBM/LGG.

Reports have uncovered that the tumor microenvironment (TME), which consists of tumor cells and many other cells, plays a crucial role in cancer progression and could significantly influence clinical outcomes (37). As a considerable component of the TME, tumor-infiltrating immune cells are associated with the growth, invasion, and metastasis of nearly all tumors (2, 6). For example, the cancer-associated fibroblasts (CAFs), fibroblasts activated by tumors, play a crucial role in generating the interstitial matrix and contributing to advanced carcinomas’ desmoplastic stroma. Accumulating evidence has demonstrated that the CAFs were associated with poor prognosis in multiple types of cancer and could promote tumor aggressiveness and immune evasion by providing a cytokine and extracellular matrix (ECM) milieu of tumors (38–41). Furthermore, the multiple immune cells recruited by progressed tumors could stimulate an inflammatory environment and promote the secretion of cytokines and chemokines, which contribute to tumor growth, metastasis, invasion and pathological angiogenesis (10–12). Herein, our result demonstrated that the GPX8 expression might be related to cancer-associated fibroblasts and immune infiltration levels in certain tumors. Our study first implied the GPX8 expression might associate with the TME, especially the immune infiltration.

Additionally, through the enrichment analyses of GPX8-related genes, we recognized the “Glutathione Metabolism”, “Antioxidant Activity” and “Antioxidant Activity” may be the potential mechanism for GPX8 to influence the etiology(ies) and pathogenesis of cancers. Reports have demonstrated that oxidative stress is a critical metabolic feature in the TME due to stromal inflammatory cell recruitment, can promote the function of CAFs (42–44). As an important gene for preventing ROS accumulation, the expression of GPX8 may contribute to this progress and then influence the clinical outcomes.

Furthermore, we confirmed GPX8 was highly expressed in GBM cells and contributed to migration and invasion. Chen et al. had reported that the over-expression of GPX8 was observed in gastric cancer and could promote the malignant behavior of gastric cancer cells (23). Another report had demonstrated the GPX8 was up-regulated in breast cancer and associated with epithelial-mesenchymal transition (24). However, our study first indicated that GPX8 plays a cancer-promoting role in GBM cells, especially in migrative and invasive phenotypes. Reports revealed that fibrosis is driven by ROS and cytokines induced by immune cells (45, 46). The complex TME may be critical for invasion and metastasis (47, 48). Interestingly, all of these characteristics above are related to GPX8. It implied that GPX8 might be a potential target for the diagnosis and treatment of cancers.

Overall, our pan-cancer analyses of GPX8 have clarified the landscape of GPX8 expression with the clinical prognosis and immune cell infiltration, which provided a predictive biomarker in pan-cancer, especially in the GBM/LGG, KIRC, KIRP and STAD. In particular, we first confirmed the up-regulated of GPX8 in GBM cells and observed the suppression of migrative and invasive phenotypes by knockdown of GPX8. Guided by the bioinformatics analysis, our study further revealed the mechanisms of the diagnosis and prognosis of cancers. However, although our findings have indicated the correlation between the expression of GPX8 and TME, more experiments are required to explore, including the role of GPX8 in cell proliferation, apoptosis and the molecular mechanisms underlying. Furthermore, the validation of GPX8 expression in more tumor samples needs to be further studied, as well as the relationship between GPX8 expression and the survival curve of patients with GBM. Moreover, as GPX8, which does not have GSH binding domain, is reported to contain a weak GPX activity, the precise biological function of GPX8 remains to be explored (21, 49).

## Conclusion

Our study showed a correlation of GPX8 expression with clinical prognosis, DNA methylation and immune infiltrates. Furthermore, we first confirmed GPX8 was highly expressed in GBM cells and contributes to migration and invasion. These results provided a predictive biomarker and an inclusive understanding of the GPX8 expression in multiple tumor types, especially in GBM.

## Data Availability Statement

Publicly available datasets were analyzed in this study. This data can be found here: TIMER database (http://timer.cistrome.org/), ONCOMINE database (www.oncomine.org), The CPTAC analysis of the UALCAN portal (http://ualcan.path.uab.edu/analysis-prot.html), The Human Protein Atlas (HPA) (http://www.proteinatlas.org/), The Kaplan-Meier Plotter (https://kmplot.com/analysis/), The PrognoScan (http://dna00.bio.kyutech.ac.jp/PrognoScan/index.html), GSE4412-GPL97, GSE4271-GPL97, GSE17536, GSE17536 and GSE31210. The ONCOMINE database is no longer available, any data request should be directed to the corresponding authors.

## Author Contributions

XT, ZY, DW, and ZR conceived the project. ZR, YH, QY, and JG performed the data analysis. BL and ZY participated in writing the manuscript. ZR and HH edited the language. All authors contributed to the article and approved the submitted version.

## Funding

This work was supported by National Natural Science Foundation of China (31660265, 31960208, 81060145, and 81560356), Youth Fund of Guizhou Provincial People’s Hospital (GZSYQN[2015]06 and GZSYQN[2019]15), Subsidy Foundation of National Natural Science Foundation of Guizhou Provincial People’s Hospital (Guizhou Science and Technology Platform (2017)5724), Science and Technology Foundation of Guizhou Province (Guizhou Science and Technology J Word (2015)2096), Subsidy Foundation of National Natural Science Foundation of Guizhou Provincial People’s Hospital(GPPH-NSFC-2019-1).

## Conflict of Interest

The authors declare that the research was conducted in the absence of any commercial or financial relationships that could be construed as a potential conflict of interest.

## Publisher’s Note

All claims expressed in this article are solely those of the authors and do not necessarily represent those of their affiliated organizations, or those of the publisher, the editors and the reviewers. Any product that may be evaluated in this article, or claim that may be made by its manufacturer, is not guaranteed or endorsed by the publisher.

## Supplementary Material

Supplementary Table 1 Primer sequences for qRT-PCR.

Supplementary Table 2 Sequences of shRNAs.
