# Supplementary material for: A Comprehensive Analysis of the Glutathione Peroxidase 8 (GPX8) in Human Cancer
Source: Front Oncol. 2022 Mar 25;12:812811. doi: 10.3389/fonc.2022.812811 (PMC8991916; doi:10.3389/fonc.2022.812811)
Supplement: Supplementary Table 1 — Primer sequences for qRT-PCR. [file Table_1.docx]

**SUPPLEMENTARY TABLES**

Table S1 Primer sequences for qRT-PCR

| Primers | Sequence (5'->3') |
| --- | --- |
| GPX8 | Forward GCCCAGAGCAAAGGTTTCACTA |
|  | Reverse CACTGGCCACGTTTACAACT |
| GAPDH | Forward ATGACATCAAGAAGGTGGTGAAGCAGG |
|  | Reverse GCGTCAAAGGTGGAGGAGTGGGT |

Table S2 Sequences of shRNAs

| shRNAs | Sequence (5'->3') |
| --- | --- |
| sh-GPX8-1 | CGACUUCCUUGACGUGUUUCU |
| sh-GPX8-2 | CCUCUUGGACGUAAAUCUAAA |
| sh-NC | UUCUCCGAACGUGUCACGUTT |
